# Association between Polymorphisms in Interleukin-17A and -17F Genes and Chronic Periodontal Disease

**DOI:** 10.1155/2012/846052

**Published:** 2012-12-06

**Authors:** Jôice Dias Corrêa, Mila Fernandes Moreira Madeira, Renata Gonçalves Resende, Jeane de Fátima Correia-Silva, Ricardo Santiago Gomez, Danielle da Glória de Souza, Mauro Martins Teixeira, Celso Martins Queiroz-Junior, Tarcília Aparecida da Silva

**Affiliations:** ^1^Departmento de Clínica, Patologia e Cirurgia Odontológicas, Faculdade de Odontologia, Universidade Federal de Minas Gerais, 31.270-901 Belo Horizonte, MG, Brazil; ^2^Departmento de Microbiologia, Instituto de Ciências Biológicas, Universidade Federal de Minas Gerais, 31.270-901 Belo Horizonte, MG, Brazil; ^3^Departamento de Bioquímica e Imunologia, Instituto de Ciências Biológicas, Universidade Federal de Minas Gerais, 31.270-901 Belo Horizonte, MG, Brazil; ^4^Departamento de Clínica, Patologia e Cirurgia Odontológicas, Faculdade de Odontologia, Universidade Federal de Minas Gerais, Avendia Antônio Carlos 6627, 31.270-901 Belo Horizonte, MG, Brazil

## Abstract

*Objective*. Interleukin-17 (IL-17) is a cytokine that induces neutrophil recruitment and the release of inflammatory mediators in several inflammatory conditions; nevertheless, the involvement of IL-17 gene polymorphisms in chronic periodontitis (CP) has not been addressed yet. Our aim was to evaluate the association between periodontal status and the polymorphisms IL-17A G197A and IL-17F C7488T in subjects with CP along with their impact on levels of inflammatory mediators. *Material and Methods*. Genomic DNA was obtained from 30 CP patients and 30 healthy controls (HCs). IL-17A G197A and IL-17F C7488T polymorphisms were determined using PCR-RFLP. Serum and periodontal tissues were collected and processed for ELISA, myeloperoxidase (MPO), and/or microscopic analysis. *Results*. The frequencies of genotypes in the CP group were significantly different from those of HC. Odds ratio indicated that increased risks for CP were associated with the -197A allele, not with the -7488T allele. In addition, the -197A allele was correlated with worse clinical parameters, higher MPO activity, and increased expression of inflammatory mediators (IL-17A and IL-8) than the other genotypes. *Conclusions*. These results indicate that the IL-17A -197A allele is associated with increased risk for CP, likely because this genotype relates to the enhanced inflammation in periodontal tissues.

## 1. Introduction

Periodontitis is an inflammatory disease that affects the tooth-supporting tissues. It is considered one of the most significant causes of tooth loss in humans and may associate with systemic diseases, such as diabetes and arthritis [1–4]. Pathogens of the subgingival bacterial biofilm are essential for the initiation and progression of periodontitis. Nevertheless, disease results from the host inflammatory reaction that primarily mediates tissue damage [2, 5, 6]. For many years, the pathogenesis of periodontitis was classically viewed as involving an immunological Th1/Th2 paradigm. According to this view, the tissue destructive Th1 cells and cytokines would arise in the early period of the disease, while the tissue protective Th2 cells and cytokines would arise in the late phase [2, 6, 7]. However, in several clinical contexts, the Th1/Th2 balance/imbalance is not sufficient to explain the progression and/or remission of periodontitis observed in patients [7].

In 2005, the Th17 subset of CD4^+^ T cells was identified [8] and added greater complexity to Th function. Th17 cells are generally considered to be proinflammatory, in particular through the production of the cytokines interleukin-17A (IL-17A) and IL-17F. These cells and cytokines have been associated with the pathogenesis of an extensive list of autoimmune and inflammatory diseases, including rheumatoid arthritis, inflammatory bowel diseases, psoriasis [9–11], and periodontitis [12–15]. The majority of studies have reported increased IL-17 levels associated with the development of chronic periodontitis (CP) [14–18]. In experimental models, Th17 is suggested to play a role in the development of disease [17]. In humans, elevated levels of IL-17 have been reported in patients with CP, but it is not clear why such elevated levels are found in these patients [14, 15, 17]. The study of genetic polymorphisms in CP has received increasing attention lately as they describe the contribution of genes to disease progression [18].

Allelic variants of cytokine genes are typically related to either higher or lower production of these molecules [18, 19]. In this regard, it is reasonable to hypothesize that genetic variation affecting the expression or activity of IL-17 may influence the susceptibility and severity of periodontitis. IL-17A and IL-17F genes are mapped on the same chromosome at position 6p12 [20], and the polymorphisms of IL-17A G197A (rs2275913) and IL-17F C7488T (rs763780) have recently been associated with higher susceptibility to rheumatoid arthritis [21] and ulcerative colitis [22]. Nevertheless, the possible involvement of IL17 gene polymorphisms in CP has not been evaluated yet. 

The purpose of the current study was to determine whether the IL-17A G197A and IL-17F C7488T polymorphisms were associated with increased susceptibility to periodontitis. We studied the association between each single nucleotide polymorphism (SNP) and the clinicopathological features of CP and local and systemic production of inflammatory mediators.

## 2. Material and Methods

### 2.1. Subjects and Sample Collection

Gingival tissue samples were obtained from periodontal tissues resected during periodontal surgery from 30 patients with CP who attended the Periodontal Clinic, School of Dentistry, Universidade Federal de Minas Gerais (UFMG). All patients had a previous history of CP and were diagnosed according to previously described criteria, including >35 years of age, no marked familial aggregation, and variable distribution of periodontal destruction [23, 24]. The inclusion criteria were subjects with attachment loss >5 mm at more than one tooth, more than three sites of probing depth >6 mm, and lesions distributed at more than two teeth in each quadrant; these subjects were diagnosed with CP. Exclusion criteria were aggressive periodontitis, use of antibiotic, anti-inflammatory and/or immunosuppressive medications in the 6 months preceding the research, and/or any systemic diseases (i.e., immunologic and autoimmune disorders, diabetes mellitus). Thirty periodontally healthy patients subjected to fully impacted third molar extraction, age- and gender-matched to the CP group, comprised the healthy control group (HC). In the current study, the individuals have not been stratified in ethnic groups based on skin color, race, or geographic origin due to the significant miscegenation among Brazilian population [25, 26].

Both groups of patients, CP and HC, were also subjected to periodontal examination including determination of probing depth (PD), clinical attachment loss (CAL), bleeding on probing (BOP), and tooth mobility. The BOP was considered positive if bleeding occurred within 30 seconds after probing [27]. Measurements were performed full mouth at 6 sites per tooth (mesiobuccal, mid buccal, distobuccal, mesiolingual, mid lingual, and distolingual). At the time of the examination, a peripheral blood sample was taken from each patient and processed for polymorphism determination and serum obtainment.

Written informed consent was obtained from all patients. This study protocol was approved by the local Institutional Ethics Committee (324/08).

### 2.2. Inflammatory Infiltrate Evaluation

Gingival tissue samples were also fixed in 10% buffered formalin, embedded in paraffin wax, and cut longitudinally (3 *μ*m). The sections were deparaffinized, rehydrated, and stained with H&E for the evaluation of the inflammatory infiltration. Inflammatory cells were counted in four fields in two independent sections (total evaluated area: ~1 mm²), using light microscope (Axioskop 40 ZEISS; Carl Zeiss, Gottingen, Germany) at 400x magnification. Data were expressed as total of inflammatory cells/field.

### 2.3. ELISA

The concentrations of the cytokines IL-17A, IL-17F, interferon (IFN)-*γ* and tumor necrosis factor (TNF)-*α*, and the chemokines CXCL10 and IL-8 were measured in gingival tissues and serum by enzyme-linked immunosorbent assay (ELISA) using commercially available kits (R&D Systems, Minneapolis, MN, USA). 

The assay was performed according to the manufacturer's instructions. In brief, tissue samples have been weighed, mechanically homogenized in buffer solution (0.4 mM NaCl, 10 mM NaPO_4_, pH 7.4) containing inhibitors of proteases (0.1 mM phenylmethylsulfonyl—PMSF fluoride—0.1 mM benzethonium chloride, 10 mM EDTA and 0.01 mg/mL aprotinin A) and Tween 20 (0.05%), pH 7.4 (normalization: 1000 *μ*L of solution for 100 mg of wet tissue), and centrifuged (10,000 rpm, 10 min. 4°C).

Each cytokine was detected by an anticytokine horseradish peroxidase-labelled monoclonal antibody. The OPD (*o-*phenylenediamine dihydrochloride, Sigma-Aldrich, Saint Louis, MO, USA) peroxidase substrate kit was used to determine the amount of horseradish peroxidase bound to each well. The reaction was stopped by the addition of 1 M sulfuric acid (H_2_SO_4_). The plates were read at 492 nm. The data were determined using a standard curve prepared for each assay and expressed as picograms of cytokine/chemokine per 100 mg of tissue or mL of serum.

### 2.4. Myeloperoxidase

Gingival tissue samples were also used for determination of myeloperoxidase (MPO) activity, a neutrophil enzyme marker, as previously described [28]. After processing for ELISA, the remaining tissue pellets were subjected to hypotonic lysis: 0.2% NaCl solution for 30 s followed by addition of an equal volume of a solution containing 1.6% NaCl and 5% glucose. After further centrifugation, the pellets were resuspended in 0.05 M sodium phosphate buffer (pH 5.4) containing 0.5% hexa-1,6-bisdecyltrimethylammonium bromide (HTAB, Sigma-Aldrich). The suspensions were freeze thawed three times and finally centrifuged at 10,000 rpm for 10 min at 4°C. MPO activity in 25 *μ*L of the resulting supernatant was assayed by adding 25 *μ*L of 3,3′-5,5′-tetramethylbenzidine (TMB, Sigma-Aldrich) prepared in dimethylsulfoxide (DMSO, Merck, NJ, USA, 1.6 mM), and 100 *μ*L of H_2_O_2_ (0.002%, v/v) was diluted in phosphate buffer (pH 5.4) containing 0.5% HTAB. The assay was performed in a 96-well microplate incubated for 5 min at 37°C. The reaction was stopped by adding 100 *μ*L of 4 M H_2_SO_4_ and quantified colorimetrically at 450 nm in a spectrophotometer.

### 2.5. DNA Isolation and Genotyping Analysis

Total genomic DNA was extracted from blood samples using QIAamp DNA Blood Mini Kit (Qiagen, Valencia, CA, USA) according to the manufacturer instructions. Quality, integrity and quantity of DNA were analyzed by Nanodrop spectrophotometer (Thermo Scientific-GE). Single nucleotide polymorphisms (SNPs) of the IL-17A (rs2275913) and IL-17F (rs763780) genotyping were performed by polymerase chain reaction-restriction fragment length polymorphism (PCR-RFLP) (Table 1).

The PCR amplification was performed in a total volume of 25 *μ*L mixture containing 100 ng genomic DNA, 1.0 *μ*M of each primer 20 *μ*L of Premix buffer (Phoneutria Biotecnologia, Belo Horizonte, Brazil). According to the manufacturer, the Premix buffer contained 50 mM KCl, 10 mM Tris-HCl pH 8.4, 0.1% Triton X-100, 1.5 mM MgCl2, deoxynucleoside triphosphates, and 1.25 units of Taq DNA polymerase.

PCR products were digested overnight at 37°C with XagI (Fermentas) for IL-17A G197A. The products of IL-17A G197A and IL-17F C7488T were viewed in a 6.5% polyacrylamide gel electrophoresis stained with silver.

### 2.6. Data Analysis

Data were expressed as mean ± standard deviation (SD). All data were analyzed using SPSS 17 (SPSS Inc., Chicago, IL, USA). Chi-square test analysis was used to test for deviation of genotype frequencies from Hardy-Weinberg equilibrium.

The levels of cytokines in periodontal tissues and the frequency of gene polymorphisms were compared by the Student's *t*-test and chi-square test. Odds ratios were calculated for the minor allele at each SNP. *P* values < 0.05 were considered statistically significant.

## 3. Results

### 3.1. Differences between Healthy Controls (HCs) and Chronic Periodontitis (CP) Subjects

The demographic characteristics of the studied population are presented in Table 2. The age and gender were not significantly different between groups. The frequency of smoker subjects in the studied sample was 3.3% in HC (*n* = 1) and 20% in CP (*n* = 6) groups. These patients did not present significant differences in the clinical parameters and production of inflammatory mediators when compared with nonsmokers (*P* > 0.05; data not shown). In this regard, data from smoker and nonsmoker subjects were grouped and presented together. 

The clinical features PD, CAL, BOP, and tooth mobility (not shown) were significantly higher in the group CP than those in the group HC (*P* < 0.0001) (Table 2). 

**Figure 1:**
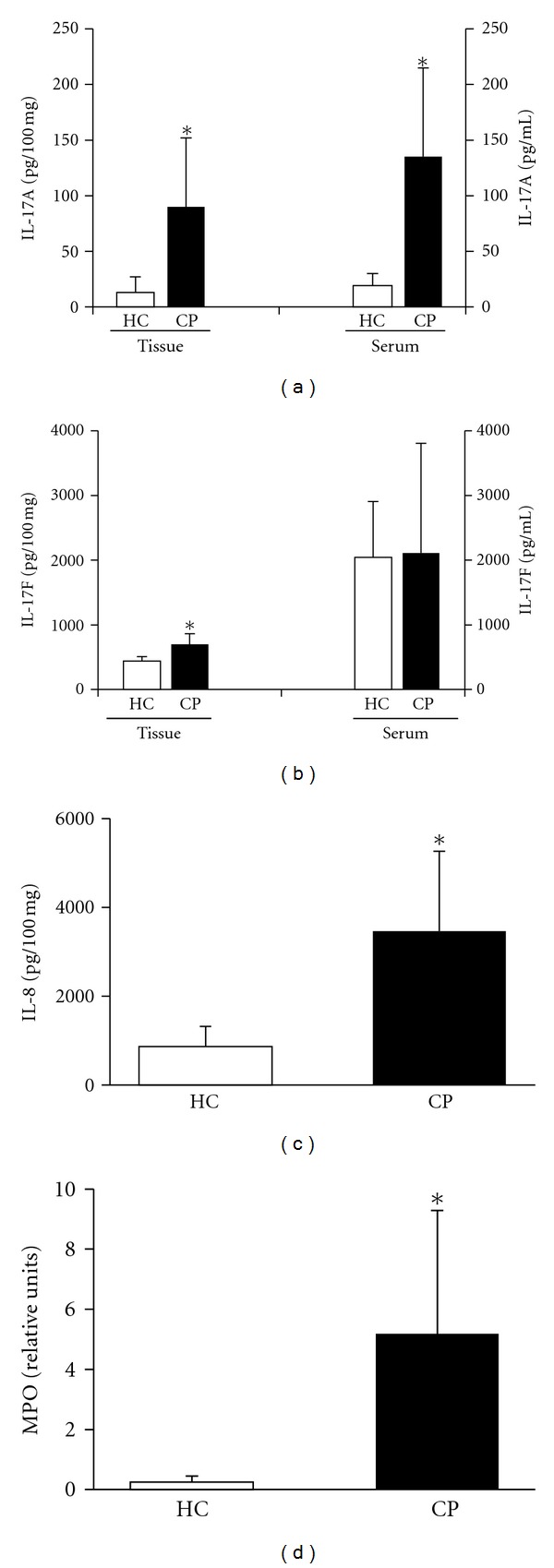
Levels of (a) IL-17A and (b) IL-17F in the gingival tissue and serum samples from CP and HC subjects. (c) Levels of IL-8 and (d) MPO activity in gingival tissue samples from CP and HC subjects. *Statistically significant difference at *P* < 0.05. HC: healthy control, CP: chronic periodontitis, and MPO: myeloperoxidase.

Besides clinical features, we also evaluated the levels of the cytokines IL-17A, IL-17F, IFN-*γ* and TNF-*α*, and the chemokines IL-8 and CXCL10 in periodontal tissues and/or serum of HC and CP subjects. Overall, levels of inflammatory mediators were increased in tissue and serum of CP patients when compared to the HC group, with the exception of IL-17F in serum (*P* > 0.05) (Figures 1(a)–1(c)). Levels of IFN-*γ* (HC: 45 ± 23 pg/100 mg tissue; CP: 131 ± 98 pg/100 mg tissue; *P* < 0.0001), TNF-*α* (HC: 21 ± 9 pg/100 mg tissue; CP: 117 ± 49 pg/100 mg tissue; *P* < 0.0001), and CXCL10 (HC: 15 ± 4 pg/100 mg tissue; CP: 34 ± 27 pg/100 mg tissue; *P* = 0.003) in periodontal tissues were also greater in CP patients than those in controls. Moreover, the inflammatory infiltrate in the gingival tissue, characterized by mixed polymorpho- and mononuclear cells, with a predominance of mononuclear leukocytes, was significantly higher in the CP than in the HC group (HC: 18 ± 8 inflammatory cells/field; CP: 88 ± 20 inflammatory cells/field; *P* < 0.0001). MPO activity was also significantly greater in CP than in HC subjects (*P* < 0.0001) (Figure 1(d)). 

Frequencies of polymorphisms (IL-17A G197A and IL-17F C7488T genotypes) were investigated in blood samples of HC and CP subjects (Table 3). The frequency of these genotypes agreed with the Hardy-Weinberg equilibrium (*P* > 0.05). The mean ages of the control group (AA: 44.2; AG: 47.8; GG: 45.2 years old) and patients with CP (AA: 40.0; AG: 42.0; GG: 40.8 years old) versus genotype did not present statistical differences (*P* > 0.05). The IL-17A genotypes of the CP group (GG 20%; GA 30% and AA 50%) were significantly different from the frequencies observed in the HC group (GG 59.26%; GA 14.81% and AA 25.92%) (*χ* ^2^ = 9.307; *P* = 0.01). The overall A carrier subjects (GA or AA) were associated with increased risk for periodontal disease when compared with GG carriers (OR 3.00, 95% CI: 1.34–6.67, *P* = 0.001). In contrast, the distribution of the IL-17F C7488T polymorphism was similar among the groups (*χ* ^2^ = 0.954; *P* = 0.62) (Table 3).

### 3.2. Association between the IL-17A G197A and IL-17F C7488T Polymorphisms and Clinical Periodontal Parameters

In view of the results indicating higher frequency of AG/AA alleles among the IL-17A G197A genotypes of patients with periodontitis, we further investigated whether some of these polymorphisms were associated with worse clinical periodontal parameters. As shown in Table 4, the intragroup comparison of the three IL-17A G197A genotypes indicated that PD and CAL, but not BOP, were significantly higher in AG and AA subjects than in patients with the GG genotype. Indeed, there was a significant correlation between the levels of IL-17A and PD in subjects with the genotype AA (data not show). In contrast, the IL-17F C7488T genotypes did not affect the clinical features of periodontally affected patients (Table 4).

### 3.3. Association between the IL-17A G197A and IL-17F C7488T Polymorphisms and Inflammatory Features

**Figure 2:**
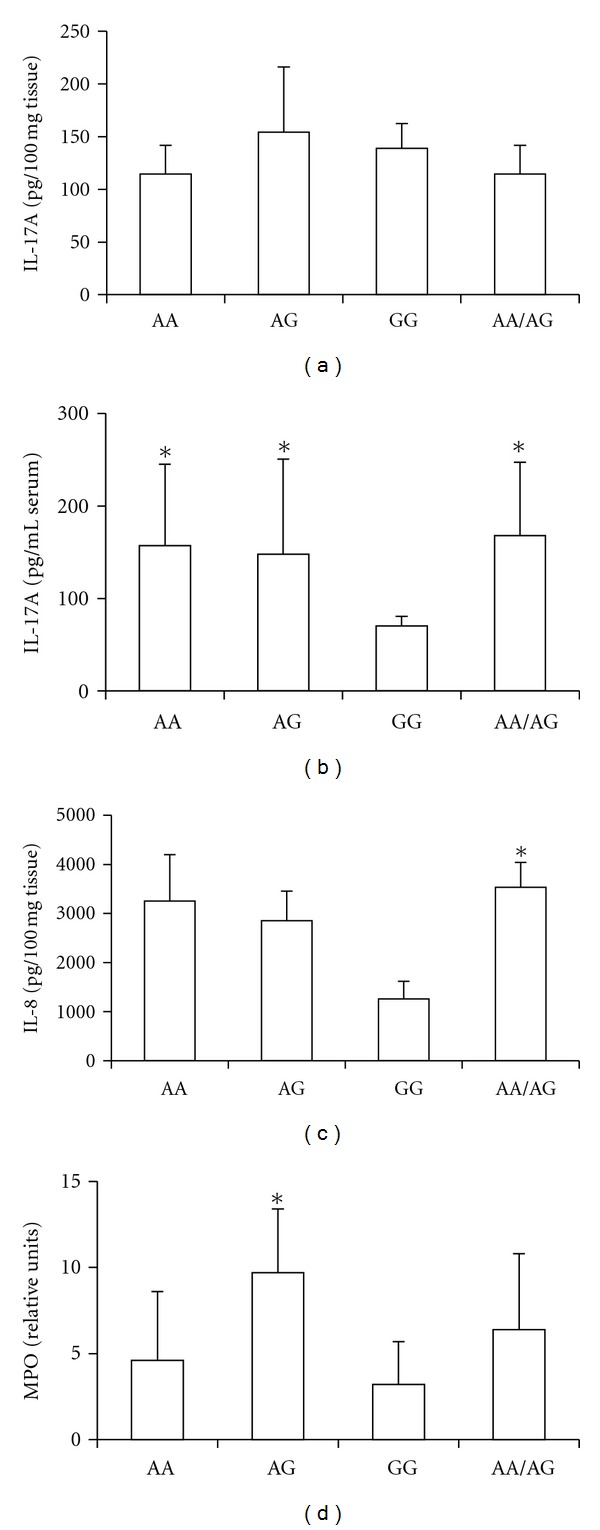
Levels of inflammatory mediators in chronic periodontitis patients according to each IL-17A G197A genotype. (a) Levels of IL-17A in gingival tissues and (b) serum. (c) Levels of IL-8 and (d) MPO (myeloperoxidase) activity in gingival tissue samples. *Statistically significant difference (*P* < 0.05) comparing with the genotype GG.

The association between IL-17 gene polymorphisms and the presence of inflammatory mediators in periodontal tissues and serum was also investigated. As shown in Figure 2(a), the levels of IL-17A in periodontal tissues from cases were not different when comparing the IL-17A G197A genotypes to each other. However, the serum levels of IL-17A were higher in subjects with the allele A than in subjects with the alleles GG (Figure 2(b)). In patients without CP, there were no differences in the levels of IL-17A in the gingival tissue or serum among the genotypes (gingival tissue: AA: 14 ± 9 pg/100 mg tissue; AG: 17 ± 17 pg/100 mg tissue; GG: 8 ± 3 pg/100 mg tissue; *P* > 0.05; serum: AA: 26 ± 10 pg/100 mg tissue; AG: 14 ± 1 pg/100 mg tissue; GG: 17 ± 10 pg/100 mg tissue; *P* > 0.05). Interestingly, the levels of the chemokine IL-8 were increased in tissues from CP patients with the allele A (Figure 2(c)), while the levels of CXCL10 were higher only in AG carriers (AA: 35 ± 29 pg/100 mg tissue; AG: 60 ± 7 pg/100 mg tissue; GG: 15 ± 2 pg/100 mg tissue; *P* = 0.004), as also occurred for MPO activity (Figure 2(d)). The histological findings indicated a mixed polymorpho- and mononuclear inflammatory infiltrate equally distributed among the polymorphisms groups (AA: 95 ± 11 inflammatory cells/field; AG: 87 ± 25 inflammatory cells/field; GG: 97 ± 5 inflammatory cells/field; *P* > 0.05).

**Figure 3:**
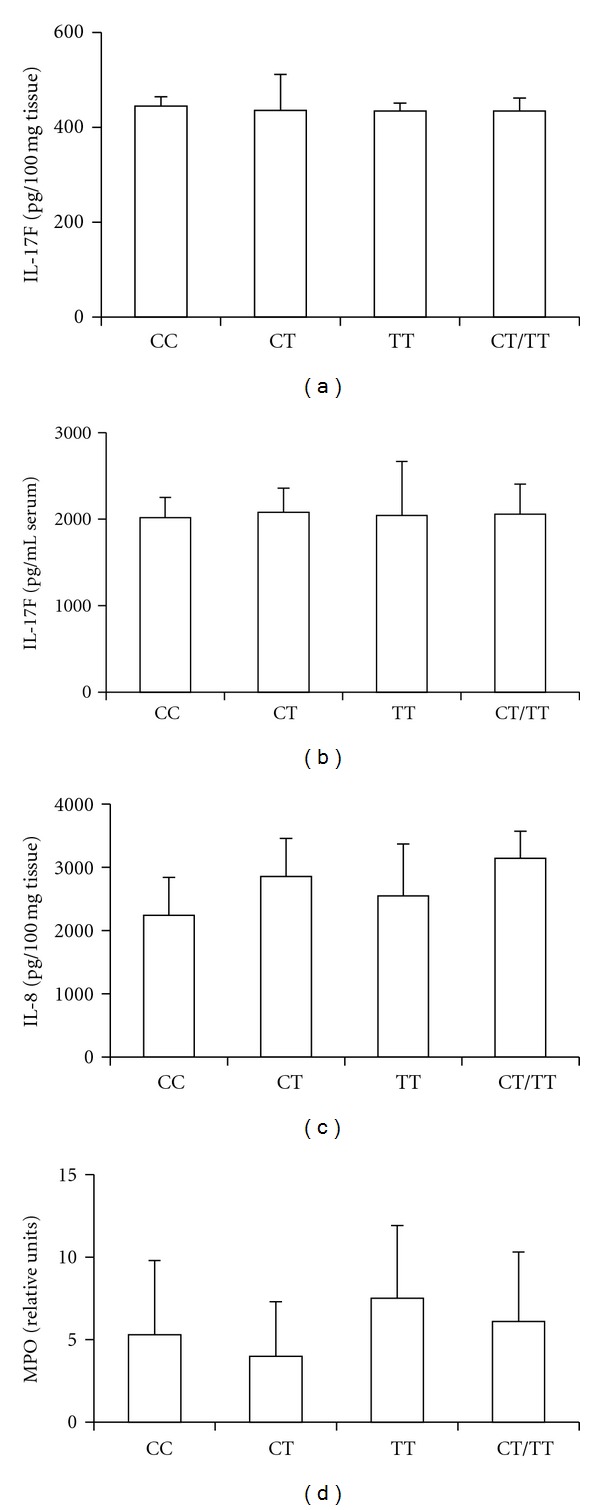
Levels of inflammatory mediators in chronic periodontitis patients according to each IL-17F C7488T genotype. (a) Levels of IL-17F in gingival tissues and (b) serum. (c) Levels of IL-8 and (d) MPO (myeloperoxidase) activity in gingival tissue samples.

The levels of IL-17F were not different, neither in periodontal tissues nor in serum, among the IL-17F C7488T genotypes (Figures 3(a) and 3(b)). The same occurred with levels of IL-8 and MPO activity, which were not different among the groups (Figures 3(c) and 3(d)). In regard to inflammatory infiltration, there were no differences among the three genotypes (CC: 102 ± 11 inflammatory cells/field; CT: 115 ± 23 inflammatory cells/field; TT: 82 ± 24 inflammatory cells/field; *P* > 0.05).

## 4. Discussion

Several experimental and clinical studies have shown that IL-17 levels are elevated in diseased human periodontal tissues and may play a destructive role in experimental models of periodontal disease [3, 14–17, 29, 30]. In the current study, we investigated the involvement of IL-17 genes polymorphisms in CP. Our results confirm that IL-17 levels are elevated in periodontal tissues of CP patients. More importantly, we show for the first time that polymorphisms of IL-17A, specially the SNP involving the allele A, are associated with the clinical and inflammatory parameters of disease. There are increased levels of IL-17A in the serum of allele A carriers, and this is accompanied by an increase of IL-8 and MPO activity in periodontal tissues.

The high levels of IL-17 in gingival crevicular fluid and periodontal tissues of patients with CP have been shown to associate with periodontal tissue damage [14, 17], but also seem to be relevant to control excessive microbial replication and, hence, disease [31]. Our study showed that the clinical parameters of CP were associated with increased levels of IL-17A and IL-17F in gingival tissues, in agreement with previous reports [14, 17, 30]. In contrast, only the levels of IL-17A, not IL-17F, were enhanced in the serum of CP patients. This is in line with studies showing increased serum concentrations of IL-17A, mainly for patients with aggressive periodontitis [1, 32]. As CP is marked by recurrent phases of remission and activation, IL-17 may be related to the destructive period of the disease given that this cytokine has already been shown to be overexpressed in active periodontal sites [33]. Along with the high levels of IL-17 in tissue and serum, the MPO activity and IL-8 levels were higher in periodontal tissues of CP patients than in healthy subjects, as well as the presence of neutrophils in the diseased gingiva. Despite the nonmechanistic nature of the current data, IL-17 has already been shown to present a prominent role in the activation and recruitment of neutrophils to inflammatory sites [16], and MPO and IL-8 have been reported to be correlated with worse clinical status of periodontitis [34, 35]. These findings suggest an active inflammatory scenario, with increased expression of IL-17 in CP patients and the probable involvement of IL-17 in the increased influx of neutrophils to periodontal affected sites.

After detecting high levels of IL-17 in CP, we analyzed the frequency of IL-17 polymorphisms in CP and HC subjects. The investigation of gene polymorphisms in CP has long been conducted, likely because they present a role in immune responses, tissue destructive mechanisms, and metabolic processes [36]. Several research groups have studied the association between CP and polymorphisms of candidate genes, including pro- and anti-inflammatory cytokines, such as IL-1*β*, IL-4, IL-6, IL-10, and TNF-*α* [36–40]. Most studies recognized the proinflammatory gene cluster polymorphisms, especially TNF-*α* and IL-1, as some of the best candidates associated with the induction and severity of CP [36, 41]. In the current investigation, the IL-17F C7488T polymorphism was not different in healthy subjects and CP patients. Despite some evidence suggesting that IL-17F may play a role in periodontal bone destruction [14] and also in the stimulation of some cytokines and chemokines, including IL-6, IFN-*γ*, and CXCL10 in inflammatory conditions [42], our data have shown that neither the clinical parameters nor the levels of inflammatory mediators in periodontal tissues were influenced by the different IL-17F C7488T genotypes. In contrast, when evaluating IL-17A, we detected a significant difference in the distribution of genotypes for the polymorphism IL-17A G197A comparing subjects with and without periodontitis. CP patients presented increased frequencies of AA and AG genotypes, and the presence of the allele A significantly increased the risk for CP. These findings are in line with previous studies demonstrating the relationship between IL-17 polymorphisms, especially the allelic polymorphic A, and chronic inflammatory diseases, including rheumatoid arthritis [21], Behçet's disease [43], ulcerative colitis [22], and gastric and breast cancer [44, 45]. They are also in line with the previously described role of polymorphisms of proinflammatory cytokine genes during CP [36, 41]. Nevertheless, it seems reasonable to remember that gene mutations alone are neither sufficient nor necessary to explain disease phenotype, although they may contribute significantly to environment and life-style parameters in the outcome of CP.

In the current study, we show that IL-17A G197A allele A carriers presented higher serum levels of IL-17A, worse clinical periodontal parameters, and increased neutrophil activity (MPO activity and IL-8 levels) when compared with the GG genotype. Although not mechanistically conclusive, these findings seem to be in line with the hypothesis of a neutrophil-mediated tissue injury associated with increased levels of IL-17A during CP, which has recently been suggested as a target mechanism for tissue destruction in experimental conditions of periodontitis associated with old age [16, 46]. Th17 lymphocytes have already been shown to be present and play a significant role in CP [47]. IL-17A can directly or indirectly (via production of chemokines) chemoattract neutrophils [48] and enhance the activity of proteolytic enzymes such as neutrophil protease and myeloperoxidase [31]. Moreover, IL-17A can stimulate the expression of bone resorption mediators, such as RANKL [49] and induce the direct differentiation of bone resorptive cells [50] and the production and release of a large range of inflammatory mediators [51], such as TNF-*α*, IFN-*γ*, and the chemokines CXCL10 and IL-8, all detected here. Indeed, a recent study showed that IL-17 can enhance CXCL10 production *in vitro* by TNF-*α*- and IFN-*γ*-stimulated human gingival fibroblasts [52] and may induce IL-8 production by gingival fibroblasts [53]. In line with these biological functions, the increased production of IL-17 seems to be predictive of tissue destruction in inflammatory conditions, such as in rheumatoid arthritis [54]. Altogether, these data point that IL-17A G197A polymorphism, especially carriers of the allele A, might be associated with increased expression of IL-17A, recruitment of neutrophils, and worse clinical conditions in CP patients.

In conclusion, this is the first study to show that the IL-17A G197A polymorphism is related to CP in a convenient sample of Brazilian patients. Although this is a relatively small sample, the presence of the allele A in IL-17A-197 polymorphism was associated with worse clinical and inflammatory periodontal parameters. It is not simple to determine in humans the mechanisms underlying the greater risk of disease in carriers of the IL-17A-197 allele A. However, our study suggests that the latter polymorphism may contribute to disease by regulating IL-17A production and, probably, the consequent release of inflammatory and bone destructive mediators. It is, therefore, suggested that IL-17A might be an interesting target for development of new therapies for periodontal disease, an assertion that needs testing in further cohorts and clinical trials. 

## Figures and Tables

**Table 1:** Primer sequences for each gene.

Primers	
IL-17A	Sense 5′-AACAAGTAAGAATGAAAAGAGGACATGGT-3′
	anti-sense 5′-CCCCCAATGAGGTCATAGAAGAATC-3′
IL-17F	Sense 5′-GTTCCCATCCAGCAAGAGAC-3′
	anti-sense 5′-AGCTGGGAATGCAAACAAAC-3′

**Table 2:** Demographic and clinical features of the studied subjects.

	HC (*n* = 30)	CP (*n* = 30)	*P* value
Age (SD; range)	40.5 (8.1; 26–52)	45.5 (8.7; 37–61)	0.97
Gender (% F)	60.86	50.00	0.44
Ethnic origin	Brazilian mixed population		
PD (SD)	2.50 (0.8)	4.52 (0.19)*	<0.0001
CAL (SD)	2.65 (0.15)	5.74 (0.17)*	<0.0001
BOP (SD)	2.0 (0.4)	31.17 (4.02)*	<0.0001

HC: healthy controls, CP: chronic periodontitis, SD: standard deviation, PD: probing depth, CAL: clinical attachment loss, BOP: and bleeding on probing.

*Significantly higher than control (*χ* ^2^ test or Student's *t*-test).

**Table 3:** Genotypes of IL-17 polymorphisms in patients with chronic periodontitis (CP) and healthy controls (HCs).

Genotype	HC (%)	CP (%)	*P* value	OR (95% CI)
IL-17A G197A				
AG/AA	40.73	80.00*	0.001	3.00 (1.34–6.67)
AA	25.92	50.00*	0.002	3.03 (1.34–6.86)
AG	14.81	30.00*	0.014	2.94 (1.24–7.00)
GG	59.26	20.00*	0.001	1

IL-17F C7488T				
CT/TT	73.26	63.4	0.310	1.30 (0.80–2.15)
CT	16.66	20.0	0.350	0.47 (0.09–2.30)
TT	56.66	43.4	0.400	0.58 (0.18–2.04)
CC	23.33	36.6	0.450	1

OR: odds ratio and CI: confidence interval.

*Significantly different from control *P* < 0.05 (*χ* ^2^ test).

**Table 4:** Association between IL-17A G197A and IL-17F C7488T polymorphisms and clinicopathological features of chronic periodontitis.

Genotype	*n*	PD (mm)	*P* value	CAL (mm)	*P* value	BOP (%)	*P* value
IL-17A G197A							
AA/AG	24	4.82*	0.012	6.00*	0.004	33.40	0.31
AA	15	4.58*	0.005	5.77*	0.01	39.45	0.14
AG	9	5.29*	0.007	6.429*	0.004	22.14	1.00
GG	6	3.30		4.70		23.75	

IL-17F C7488T							
CT/TT	19	4.86	0.11	5.571	0.14	25.89	0.27
TT	6	4.91	0.20	5.63	0.16	25.24	0.25
CT	13	4.67	0.18	6.67	0.59	28.30	0.71
CC	11	5.25		6.25		37.33	

PD: probing depth, CAL: clinical attachment loss, and BOP: bleeding on probing.

*Significantly different from GG or CC genotype (*P* < 0.05, Student's *t*-test).
